# The Third Regular Semi-Annual Meeting of the Illinois State Dental Society

**Published:** 1867-03

**Authors:** 


### Proceedings of Societies.

### THE THIRD REGULAR SEMI-ANNUAL MEETING OF THE ILLINOIS STATE DENTAL SOCIETY.

The Third Regular Semi-Anuual Meeting of the Illinois State Dental Society held its sessions in Chicago, commencing November 13, 1866.

The meeting was called to order by the President, Dr. H. N. Lewis, of Quincy.

The first business in order was the reading of an address by Dr. M. S. Dean, of Chicago.

The speaker, after a brief introduction to his remarks, spoke at some little length of the progress made by the profession during the last few years. No better proof of this progress could be had than a comparison between the mechanical work of the past with that of the present. The clumsily carved blocks of ivory, which, a few years ago, served the purpose of teeth, and though infinitely inferior to the work of the merest tyro in the profession of to-day, were, even in the present generation, considered to embody all the skill and art possessed by members of the profession. One of the main, though probably indirect, causes of this advancement, was the fact that a change of climate, and the replacement of the simple diet of by-gone days by the luxurious viands of recent times, has called the labors of the Dentist into more general requisition, causing him to embrace every opportunity for improvement. But, even appreciating the great progress which has been made, there is still a call for an increased display of energy and ability. Great good would be achieved by the erection of a higher standard of education. The profession required as extensive a course of study as medicine or surgery. It must improve all the aids that science affords. The education of the student should be substantial in its nature, and threefold in its character, embracing the instruction of the mind, the hand, and the eye. That is, it should be scientific, mechanical and artistic. The whole system should be well known by the practitioner, inasmuch as a knowlege of the entire system is requisite for the successful treatment of any of its principal organs. A certain acquaintance with medicine should, therefore, be the ground-work of the profession. The hand of the Dentist should be trained in the mechanics of the profession, and in the handling of instruments dextrously. It must construct well and readily what the mind conceives. The eye, by education, must be rendered acute and delicate. In noticing the general and satisfactory progress of the profession, it was observable that, during the past four or five years, that advancement had not been as general as during the five years immediately preceding. This was probably owing to the fact that the introduction of vulcanized rubber as a base caused the work of inserting teeth to be conducted so cheaply that the onward progress of the profession was somewhat checked. It was satisfactory to witness that to this general rule there were honorable exceptions, and that many members have ever continued to devote their best talents to the advancement of this important branch of surgery.

In conclusion, Dr. Dean spoke of the benefits arising from such associations as the Illinois State Dental Society. They were useful because they afforded opportunities for the interchange of ideas. Those who fail to avail themselves of the advantages of such comparative knowledge, will find that in a short time they will be outstripped by others who commenced with a smaller scientific foundation, but who have acquired knowledge from the experiences of older members of the profession. To bring about this good, the Illinois Society was established, and in considering the benefits which will accrue from a free and full interchange of experience and ideas, its members should relate their respective practices, their successes, and, if they have any, their failures. While the Dentist should study to become eminently useful in his profession, he must not neglect the collateral sciences. There is a great danger that in the every-day routine the cramped mind will become confined to its narrow limits, and lose the rich and mellow fruit of science and literature.

Dr. J. Ward Ellis moved that during the absence of the Secretary, Dr. L. P. Haskell be appointed Secretary *pro tem.*

The following gentlemen were balloted for and declared to be duly elected:

Dr. I. D. Kilbourne, of Chicago; Dr. V. R. David, of Sandwich; Dr. S. Abbott, of Wilmington; Dr. S. M. Swain, of Aurora; Dr. T. F. Woodbridge, of Mendota; Dr. George Salter, of Joliet.

### CONSTITUTIONAL AMENDMENT.

Dr. J. Ward Ellis moved to so amend the by-laws of the Society as to do away with the November meetings and constitute the annual meeting, holden on the second Tuesday of May, the only regular meeting of the year.

Under the provisions of the Constitution, the motion was laid over until the next regular meeting.

### AFTERNOON SESSION.

The Society met in the afternoon, pursuant to adjournment, the President in the Chair.

### PROFESSIONAL ELEVATION.

As a commencement of the regular discussion of the meeting, Dr. J. Ward Ellis spoke at some length on the necessity of elevating the Dental profession. That profession was but a new one, but it had acquired the position of a distinct branch, and was regarded by the public as one of importance. This confidence reposed in it by the people must not be abused by the profession. Dentists needed to cultivate a higher order of education. A man must not expect to leave the mechanic's bench, and commence the practice of Dentistry with no further aid than that afforded by a manual on the subject. He must know that the highest qualifications are necessary, and then when he feels competent in his profession, he must set his mark high. Every man should aim to excel in operation rather than attempt to compete with others in the matter of low prices. This subject of competition might have been entertained twenty years ago, but it cannot now, and the man who does his work best will be the man who succeeds. Even now the popular mind has not become sufficiently schooled to fully recognize the merits of Dentistry; but, in a measure, this is perhaps one of the faults of the practitioner, and would be obviated in a great measure if all would attempt to elevate the profession rather than to build up individual practices at the expense of the reputation of some brother practitioner. In conclusion, the speaker dwelt upon the necessity of honesty of sentiment. What a man does not know, he should acknowlege boldly, rather than attempt to make some reply which can never be satisfactory, and which will frequently only prove injurious to the practitioner.

### DISEASES OF THE TEETH.

Dr. Cushing, of Chicago, next read an essay upon the causes and prevention of diseases of the teeth. He defined the diseases to be of two characters—predisposing and approximate. To fully discuss the first class of diseases, the investigator must go back to the period of gestation. Without taking into consideration the theory of hereditary transmission, it was a fact well established that the health of the mother during the period of pregnancy has a great effect upon the health of the child.

The want of proper nourishment on the part of the parent at this time, is much felt by the unborn child, and in no organs more perceptibly than in the Dental ones. Predisposing causes may also occur after dentition has been completed. Every depraved condition of the system will exercise its influence more or less upon the teeth. The approximate causes of disease may be considered solely attributable to the action of acids. These, by the exercise of a chemical process upon the teeth, destroy their structure. In speaking of the means of prevention, the speaker said that the prevention of the predisposing forms of disease rests, in a great measure, with the mother of the unborn infant. Mothers cannot place too much value upon the necessity, during pregnancy, of having a proper diet—especially food rich in the bone-making material—and in taking suitable exercise.

The reason why the teeth of people of later days are more decayed than were those of past generations, is, that we are now farther from Nature's simple plan. Indeed, the terrible condition of the present generation may be said to be one of the penalties of civilization; and the decayed condition will increase in exact ratio with the luxuriousness of the diet. Dentists should discourage the general use of pickles, lemons and other acid substances, and bring their patients as near as possible under the influence of the hygienic law. Another great cause of disease by the production of acids, is the fermentation of food lodging in the teeth after eating. The remedy for and preventive of this is cleanliness, absolute cleanliness. The Doctor recommended the use of antacids with the brush. Solution of carbonate of soda, lime water, &c., could be advantageously used three times a day, for an indefinite period.

After some discussion upon Dr. Cushing's essay, the Society adjourned until morning.

### SECOND DAY'S SESSION.

The Society met according to adjournment.

The minutes of the last regular meeting were read by Dr. L. P. Haskell, Secretary *pro tem.*, and on motion, were approved.

### ELECTION OF MEMBERS.

The following gentlemen were balloted for and duly elected members of the Society:

Dr. Thomas E. Fahnestock, of Aurora, and Dr. A. C. Allen, of Joliet.

### DECIDUOUS TEETH.

Under the topic of deciduous teeth, Dr. Kennicott spoke at some length on his experience in capping the nerves. He believed it to be a most successful manner of proceeding, and commended it as a humane and gentle mode of treatment. He condemned heroic treatment as unworthy of the humane character of the profession.

### CAPPING.

Dr.Ellis believed in heroic treatment, when by heroic was meant active treatment. For ten years he had abandoned capping nerves, except in a few cases. His experience in capping nerves had been unsuccessful, and he had come to the conclusion that he could not preserve deciduous teeth by capping in five cases out of fifty. When he practiced the method of capping, he took a small cup of platina or gold, and, after indenting it, put into it the gutta percha, and then pressed it down upon the nerve in order to get an impression. Generally he failed, but even when he did not, could not but feel that he had placed upon one of the tenderest little bundles of nerve a vegetable foreign substance which would cause inflammation and suppuration to ensue. At present, in the treatment of deciduous teeth, he steeped a pledget of cotton in creosote, and then forced it down in the cavity previous to filling.

Dr. M. W. Sherwood had met with the greatest success in the treatment of deciduous teeth by filling with amalgam. He found it expeditious and successful. In cases of the exposed nerves of children, he removed the soft portion of the dentine, and laid over the nerve a piece of bibulous paper saturated with creosote; then he filled with amalgam. The speaker had long since abandoned the practice of treating pulps by capping.

Dr. Kennicott said that in his method of capping, he always liquified the gutta percha by heating it over a spirit lamp, before he attempted to take an impression. He had tried the oxychloride of zinc, as recommended by Eastern practitioners, but did not like it as well as the liquid gutta percha.

The discussion was further participated in by Drs. Noble, Reber and others, after which the meeting adjourned until afternoon.

### AFTERNOON SESSION.

At 3 o'clock the Society met, pursuant to adjournment, the President in the chair.

The discussion of the mode of treatment of deciduous teeth having closed, the President stated that the next topic for consideration was the

### TREATMENT OF SIX-YEAR-OLD MOLAR TEETH.

Dr. M. W. Sherwood, the first speaker upon the subject, dwelt upon its great importance, and the necessity of impressing the minds of parents with the idea that they must teach their children to secure the perfect cleanliness of their teeth. He did not favor the practice of extirpating the nerves.

Dr. J. Ward Ellis believed in the great importance of the subject under discussion, and considered that it merited the close attention of the Dental profession. Men were too apt to lose sight of the importance of the treatment of six-year molars, or else to attach themselves firmly to the working out of some peculiar theory. He did not think that the proper method of treatment could be stated as a method to be followed in all cases without deviation. For example, he would not say that he would always extract or always preserve the teeth, but would be governed in his action by the circumstances of the case.

Dr. Honsinger believed that a question upon which so many were ignorant should receive the closest attention of the profession. It was the common belief with parents, that the six-year molars would be replaced by others; that they were only the first growth of teeth. Consequently, they paid but little attention to them. This idea was certainly wrong; for the fact was, that it was necessary to most carefully treat the six-year molar teeth. It was hardly practicable to define a mode of treatment which would be most successful in all cases, but the circumstances in each instance should be closely considered.

After remarks by several other gentlemen present, the discussion of the question was closed.

### THE HARD RUBBER QUESTION.

The Chair announced that Col. Fisher, the attorney whose services had been retained by the Dentists in the Goodyear Vulcanized Rubber Question, was present, and could probably give the Society some information in the premises.

Col. Fisher then addressed the meeting. From his report it appears that he had been secured to fully investigate the hard rubber patent, and ascertain the advisability of further contesting its validity. He found that some time ago the Goodyear Dental Vulcanized Rubber Patent Company commenced an action at law against a certain Dentist for an alleged infringement of their so-called patent right. In the lower Court in which the suit was instituted, the case was decided in favor of the complainants, and the question now was, whether it would be advisable to carry the case by appeal up to the Supreme Court of the United States. The investigation of the speaker had proved that the so-called invention had been first made by a Parisian Dentist many years before a patent for it was taken out in the United States by another person, who supposed himself to be the original discoverer. The Parisian Dentist had been adverse to patenting, or even publishing, the invention.

After the subject had been discussed at some length by many gentlemen present, Dr. Allport moved that the action of the Ohio Dentists, in relation to this contest, be adopted by the Illinois State Dental Society.

The motion prevailed.

### THIRD DAY'S SESSION.

The Chair announced that the subject of the treatment of six-year molars was still before the meeting.

Dr. Ellis moved to close the discussion on that subject, and pass to the consideration of the next in order.

The motion prevailed.

The Society then proceeded to consider the question of

### FILLING TEETH.

Dr. Wilson believed that saving the natural teeth was the true sphere of the Dentist. It is the first thing the student thinks he knows, but really the last thing that he certainly knows. It is a branch of the science that is not thoroughly appreciated by the public, who even yet have not learned that natural teeth, even if a little irregular, are preferable to porcelain; and that many difficulties commence with the false teeth.

The great desideratum to be sought is to perfect a filling which shall be perfectly air and water tight. He believed that almost generally there was more in the manner than in the matter of working. Regarding the material, the Doctor believed most undoubtedly in the pre-eminent virtue of gold. There may be isolated cases where, in certain hands, other materials are preferable; but in the vast majority of instances the gold is by far the best.

In the work of filling, too much attention cannot be paid to the preparation of the cavity. It must be perfectly clear, and all extraneous substances must be removed.

In the use of amalgam, the operator cannot be too careful in the preparation of the material. All the mercury that is not absolutely necessary should be squeezed out and removed. He would not finish an amalgam filling at one sitting. In respect to the manner of spreading teeth, the speaker has always used the hickory wedge, and had found it to be successful. In conclusion, Dr. Wilson summed up the work of filling in the following sentence: "First make every part of the cavity so as to be seen by the operator, then clean thoroughly, fill surely, and finish beautifully."

### THE MATERIAL FOR FILLING TEETH.

Dr. J. Ward Ellis used the alder wood as a wedge, and drove it home thoroughly near the gums. He avoided filing as much as possible. The speaker believed in the advantage of seeing the whole of the cavity, but in certain cases this could not be done; and he had met instances when he could not see the cavity at all, and had to fill by the aid of a dam to stay the saliva. In the matter of the material for filing, the speaker agreed with Dr. O. Wilson that gold was the best, but considered there was some room for discussion in the matter of the kind of gold to be used. He was in favor of non-adhesive gold, believing it to be superior to other kinds. In certain cases, as in finishing, he used the adhesive foil, but believed that ten to twenty per cent. more successful operations of filling were accomplished with non-adhesive foil, than by the use of the adhesive, or sponge foil. With their use there was more difficulty in packing to the wall of the cavity, and the anchoring points were more likely to fail. In relation to the non-adhesive foil, the speaker considered it was easier to make a successful filling with it, and less skill and science were required at the hands of the operator. In building out the form of the tooth, the speaker admitted that he frequently used the adhesive foil; and in conclusion, he spoke of two operations recently performed by him, in which he built up the walls of some molar and bicuspid teeth, some of whose walls were still remaining, by the use of the adhesive gold.

### CRYSTAL GOLD.

Dr. Honsinger had been in practice as a Dentist for about nineteen years. During about half that time, he had used the non-adhesive foil; but his greatest successes have been attained by the use of crystal gold. This fact may be due somewhat to his increased experience, but in the main he believed it was due to the superiority of the material. His opinion was that more gold in the crystalline form can be introduced into the cavity than by the use of foil. The crystallized gold can be packed more firmly than the foil, and the best filling is certainly that one which excludes most perfectly the air and water. In relation to the matter of filling, the speaker had, in his earlier days, lost many teeth by a fear to use the file. Many failures in approximate and crown fillings have occurred, for the reason that the cavity is too small to allow a perfect success. The speaker always endeavored to get a full look into the cavity to be filled, in order to know well how to proceed in the operation. Though not an advocate of indiscriminate filing, he used the file extensively. Formerly he used a flat file, but he has since found that the most effective instrument is of the V shape.

Such a file is especially successful in working on bicuspid teeth.

In relation to the filling of teeth with crystal gold, Dr. Honsinger said that he did not know that he failed five times a year. He protested against the spreading of teeth by forcible wedging. Sometimes it may be well to exercise a moderate strain, but the practice of forcible, sudden wedging was not advisable, in the speaker's opinion. In spreading teeth, he always used virgin rubber. This rubber he cuts into small strips of various thicknesses, and inserts between the teeth to be spread. By commencing with the thinnest strip, and continuing to replace by thicker ones, he always succeeded, in a week or eight days, in spreading the teeth sufficiently to commence filling. In the matter of front teeth, the speaker seldom used a file, except the tooth was so decayed as to render the edges ragged and irregular.

### FILING TEETH.

Dr. Kilbourne, of Aurora, believed that the use of the file was necessary, and that it had saved many more teeth than it had destroyed. In the matter of wedging, the speaker had used cotton, with great success. It was less painful to the patient, and succeeded in spreading the tooth as effectually, and in about the same time, that strips of rubber would. He considered that the two great causes of failure in the filling of teeth with gold are, first, the want of cleanliness in the cavity at the bottom; and, secondly, the want of a thorough packing of the gold at the base and sides of the tooth.

### SPREADING TEETH.

A gentleman present related his experiences in spreading teeth by means of a ligature of cotton twine tied around the tooth. He considered the method a good and effective one.

### NECESSARY PAIN.

Dr. Allport acknowledged that the use of the wedge was frequently painful, but believed it was the duty of the Dentist not to allow this fact to deter him, if he believed the operation would result in the future good of the patient. Better cause a little instant pain, to produce comfort hereafter. If pain is absolutely necessary to perfect an operation, the practitioner ought not to mind inflicting it. He believed in wedging thoroughly, and considered that the act was really a preventive of pain, inasmuch as the pressure at the apex of the root in a measure paralyzes the nerve, causing much less pain during the subsequent work of excavating. If the tooth is loose, the wedge steadies it, and, by preventing it from swaying about, saves much inconvenience and pain.

Dr. Kennicott, in spreading teeth, used a pledget of cotton saturated with some sedative. It accomplished all that the rubber strips or wooden wedge did, and without pain. If he used a file, he was careful to apply an anæsthetic. If he used a file, he believed it should be used "heroically" and thoroughly, but he deprecated its use at all as unnecessary and painful. When compelled to use a wedge, he chose the softest pine wood, and then selecting one about one-third larger than the cavity into which it was to be introduced, he pushed it in between the teeth with a pair of pliers. In conclusion, the speaker earnestly spoke against operations which inflicted pain upon the patient. He considered that no Dentist who "had been through the mill" and suffered the pain of some heroic operation himself, would inflict pain upon a patient, if he possibly could avoid it.

Dr. Allport contended that operations could not be performed without pain, and that it was useless to consider such a thing practicable. He deprecated the education of the popular mind with the belief that Dental operations could be performed without personal discomfort and pain.

Dr. Ellis believed that the wedge did not produce more pain than other appliances, and considered its use necessary.

The meeting then adjourned until 3 o'clock in the afternoon.

### AFTERNOON SESSION.

In the afternoon the Society met, pursuant to adjournment, the President in the chair.

The Chair announced as the subject for discussion,

### THE TREATMENT OF EXPOSED NERVES.

Dr. Kilbourne, of Aurora, mentioned several cases which came under his own attention. He applied arsenic, and then at the request of the parties proceeded to fill the teeth immediately. The teeth were principally bicuspids and molars. He left the arsenic in the tooth and filled over it. He was not an advocate of that mode of practice, but in that instance tried the experiment, and found it successful. His general practice was, when he found an exposed nerve, to first extirpate it, and then, as soon after as practicable, commence to fill the cavity.

### DESTRUCTION OF THE NERVE.

Dr. Honsinger believed the question had been thoroughly investigated at former meetings. In the treatment of pulps, he had long abandoned the practice of capping. Generally, when he found a pulp wounded so as to produce blood, he destroyed it. In destroying the nerve, he used the arsenical paste. In the case mentioned by Dr. Kilbourne, no serious results appear to have ensued by leaving the arsenic in permanently, but the speaker always desired to remove the paste as soon after the nerve is destroyed as possible. He then removes the pulp, and shortly afterward commences filling; but fears to commence filling immediately after destroying the nerve, inasmuch as ulceration might ensue. He would not fill the tooth until all soreness and inflammation had been removed. In some cases he could not subdue the inflammation and then would not fill until the tooth was sufficiently healthy to permit the patient to bite. The Doctor had found the application of cotton steeped in creosote before filling, advantageous and successful.

Dr. M. S. Dean thought the treatment of exposed nerves the most important subject that had been presented for discussion. His experience in capping had been that of almost every other Dentist—unsatisfactory, and had long since abandoned it. His usual practice now was to destroy with creosote and arsenic, although in some cases where in excavating he had found the nerve slightly exposed, and the patient young and vigorous, he had applied creosote or perchloride of iron and immediately filled the cavity. Although this practice had sometimes been successful, it was so uncertain that he seldom adopted it.

He considered the saving of the pulp of so much importance that he hoped the scientific minds of the profession would be concentrated upon that *vital* point until the saving of them alive should become as common as their destruction was now general.

He had seen a few teeth upon which the operation of excising a portion of the pulp had been performed by Dr. Allport. I had no doubt but that the nerve was covered with ossific matter, but recovered its healthy condition. He had examined these cases from three to nine months after the operation had been performed, and from what he had seen was fully satisfied in his own mind that these operations could be and had been successfully performed. He had never attempted the operation himself, but should when a favorable opportunity presented itself, as in case of failure he should only accomplish accidentally what he had otherwise done intentionally.

Dr. Kennicott, as a rule, found no rule in treating simple cases of exposed nerve. He believed in giving the nerve a fair trial before scalping it, gouging it out, or destroying it with arsenious acid. He believed the bounden duty of every operator was first to attempt to preserve the vitality of the pulp. After applying some sedative to the nerve, the speaker believed in capping it. They must allay irritation, inflammation, or all that could produce it; then, after cleaning the cavity, the cap should be prepared. Its lining should be of some non-conducting substance, to exclude air or moisture. If subsequent inflammation ensued, the nerve need not be destroyed. The operator should then drill through the filling, when after perforating the cap, he can excavate a small portion of its lining, and in a large majority of cases effect a remedy. To attain success, there should be no vacuum beneath the cap. The space should be wholly filled, and until it is filled by some proper substance, the work can not be complete. The caps he conveniently cut out of gold, and these caps may be kept on hand, though the lining should not be placed in until the moment when it is required to be used.

Dr. Kilbourne, of Aurora, opposed the practice of capping. He did not believe it was practicable, and had abandoned it long ago.

Dr. Sherwood, of Chicago, said: A gentleman came to me with the nerve exposed in a lower wisdom tooth. On examination, I thought it so favorable a case to try Dr. Allport's treatment, and the patient had had such a bad experience with devitalized teeth, that I advised him to go to Dr. Allport and let him treat it. He did so, and the tooth was filled with Hill's stopping. It gave no pain; subsequently, was used three or four weeks, when some Dentist having expressed the opinion that it was dead, Dr. Allport removed the filling. He found the nerve living and healthy, and the orifice filled with a white lymph, and all right. In refilling, the nerve was a little pressed, and some grumbling was felt for some days, but it is now well and promises to be lasting.

The next case was a young lady for whom Dr. Allport had treated and filled the first right superior molar, eight months previously. She had experienced no pain whatever since the operation; but being about to visit Europe, the Doctor removed the filling to see how the pulp was getting on, and called M. S. Dean and myself to see the case in that condition. There was a formation of bony substance filling the orifice of exposure, much resembling the new bone we sometimes see in pulp cavities of deeply-worn teeth, in persons of advanced life. I examined the substance with a watchmaker's glass, and felt it with an instrument.

The next case was in the mouth of the same person first alluded to—a large cavity on the side of the opposite wisdom tooth on the same jaw. The tooth had been filled with amalgam for ten or twelve years; occasionally falling out, it had been replaced. I had replaced it myself once or twice, and at this time was deepening the retaining channel when my instrument uncovered the nerve without giving the slightest warning, or even wounding the nerve. I folded a piece of bibulous paper, and placing it carefully over the nerve, filled with Lawrence's amalgam, rather soft. Four or five days after, the patient returned, with tooth aching badly. I went with him to Dr. Allport's office, and assisted in the operation. On removing the filling, two or three drops of blood gushed from the pulp, and the pain ceased. Dr. Allport gave him chloroform, and excised the nerve pulp freely. It seemed to me as though he cut out half the pulp. That tooth has only been filled with cotton saturated with arnica. At times it is a little uneasy. It is now two months or more since the operation, and our curiosity is excited to see how long the nerve will live in this condition.

These are simple facts, and as such are worth more than all theories and objections to the contrary.

In surgery, we have operations amputating, excising and wounding the nerves, and even removing portions of the brain itself. Reasoning from analogy, why may we not excise the pulps of teeth? Only by experiment and careful observation can we decide the matter. The operation is not difficult to perform. I have operated upon two cases, which are going on satisfactorily.

Dr. Allport, in regard to what Dr. Kennicott had said in regard to drilling through a filling to heal an inflamed pulp, considered it contrary to correct theory and good sense. What is the cause of inflammation, when the pulp is not wounded? Pressure, or the presence of some uncongenial foreign substance. The act of drilling through the filling cannot be performed without pressure, which, when it reaches the pulp, will force more or less of the gutta percha or spicula of bone into the pulp, which will of necessity produce irritation, keep up inflammation, and death will ensue; and furthermore, he deemed it utterly impracticable to drill through the centre of a filling with any certainty of not wounding the pulp.

In relation to the excising of exposed pulps, he would say a few words. He would protest against the use of slang phrases in these discussions, such as "scalping," "gouging," &c., as unprofessional and unbecoming a body of men who had met to discuss practical and scientific questions; and we owe it to ourselves and the profession to make use of such language as that we shall not be ashamed to have others read.

In his treatment of exposed pulps, he had not always been successful by excising a portion of it, neither did he attempt it in all cases. He trusted, however, that others might improve upon his method, and be even more successful than he had been. He had some cases of two or three years' standing, where the pulp was in a fine, healthy condition, but there was no deposit of dentine; but in most cases he had treated by this method there was a deposit of dentine.

His mode of treatment was as follows: He takes an instrument made for the purpose, and excises a portion of the pulp, leaving it in a "V" shape; then dissects it from the walls of the cavity, and induces the mouth of the wound to close by gentle pressure, or the natural resilience of the parts; saturates bibulous paper with calendula or other sedative, and lays over the pulp, and covers with cotton, and after a few days, if the pulp seems healed and in a healthy condition, covers again with three or four thicknesses of bibulous paper, and fills with "Hill's stopping," until it is ready for the permanent filling.

Dr. Kennicott took exception to the remarks of Dr. Allport, who had stated that he (Dr. K.) claimed to be able to drill through the filling and pulp in order to allay secondary inflammation.

Dr. Allport wished to interrupt the gentleman. I said that in drilling through the filling *to* the pulp the point of the instrument would wound it, and force the gutta percha and spicula of bone into the pulp.

Dr. Kennicott. I *never* wound the pulp in drilling, and the man must be a very bungling operator who would do so.

Dr. Allport. I fear, then, that most operators would be bunglers, for we are warned of our approach to the pulp by its giving pain, as it does not hurt till it is pierced, and then the job is done.

Dr. Kennicott said he performed the experiment every day in his professional life, and did not consider it senseless or dangerous to the life of the pulp, and a man of Dr. Allport's skill could do the same thing.

Dr. Allport. No I can't.

Adjourned.

### FOURTH DAY'S SESSION.

The minutes of the previous meeting were read by the Secretary *pro tem.*, amended and approved.

The Chair announced that the subject of the treatment of

### EXPOSED NERVES

was still under consideration.

Dr. Allport, without desiring to discuss the question at any further length, and in moving that the subject be passed, suggested that the term "exposed nerves" be not used. He considered the words "exposed pulp" were more suitable and expressive.

The subject was then passed, and the Society proceeded to the discussion of

### MECHANICAL DENTISTRY.

Dr. M. S. Dean suggested that under this head the Society should limit its consideration to the subject of artificial dentures, and not include the discussion of the irregularities of teeth.

The Chair announced that the question of irregularities of teeth formed the next subject for discussion, and therefore would not be considered at present.

### ALLEN'S CONTINUOUS GUM.

Dr. Dean said there was one way of inserting artificial teeth, and, in his opinion, but one way; that was, by the use of Allen's Continuous Gum. The subject, then, was entirely under the control of the Dentist, and afforded him full scope for the display of all the artistic skill he possessed. If he was an artistic operator, he would, by the use of this gum, succeed to the satisfaction of himself and patient. Vulcanized rubber is certainly useful, and perhaps practically is equal to any other substance, but the difficulty and objection is that sets cannot be inserted on rubber to look as natural and life-like as with the continuous gum. Some practitioners, certainly, by the exercise of great skill, succeed in the production of work on rubber of a very beautiful character; but, as a rule, the substance is by no means as satisfactory or successful as the first-named material.

### RUBBER AS A BASE.

Dr. Haskell did not exactly agree with Dr. Dean, that

Allen's Continuous Gum is the "only thing" though he conceded that it was certainly the most satisfactory. He acknowledged that, while in many cases the hard rubber base proves very effective, as a general rule the arbitrary character of the teeth cannot be avoided. In regard to the continuous gum, the teeth can always be placed just where they are wanted, and the gum can be arranged to suit the taste and will of the practitioner.

In the case of partial lower sets, the speaker believed in the excellence of rubber as a base. In some instances he always used a gold band in union with the rubber, and found it gave the best adaptation. In partial upper sets he used gold.

### CLASPS NOT DESIRABLE.

In reply to a question by Dr. Ellis, Dr. Haskell said that he avoided the use of clasps whenever possible. In some cases, from the articulation of the lower teeth, a clasp is necessary, but as a general thing the use should be avoided. In partial sets, the speaker found the atmospheric pressure to be quite sufficient. Such a pressure generally would last as long as the plate lasts. In some cases the sets used to sustain the teeth without any pressure at all; and of course in such instances no pressure need be applied.

### PARTIAL SETS.

Dr. Noble coincided in the belief that gold plate was the best base for partial upper sets, but considered rubber to be preferable in partial sets in the lower jaw. In such cases he would, however, frequently unite the gold with the rubber, and use a gold band, as spoken of by Dr. Haskell.

### WHERE RUBBER MAY BE USED.

Dr. J. Ward Ellis considered that, as a rule, the construction of partial sets on the rubber base, should be discountenanced by the profession. If a patient retained the four molars in the upper jaw, the speaker would not object to set the balance on the rubber base; but if a canine, an incisor, and two bicuspids were absent, he would not use such a base for any consideration. If the operator could set six or eight teeth all together, the rubber might be used, but when one tooth had to be inserted here and another there, with sound teeth intervening, the rubber base was not strong enough. In such cases, the speaker believed in the superiority of gold.

ENGLISH RUBBER.

In reply to a question by Dr. Haskell, Dr. Ellis said that he had set about three or four complete sets of teeth on the English rubber, during the past two years. So far the results had proved satisfactory, and he had not discovered anything against the base.

AN ELECTION.

Dr. Allport thought that the Society had been certainly remiss in allowing a well known Ohio practitioner to be with them for two days unnoticed. He moved that the rules be suspended in order to elect Dr. Keely, of Ohio, an honorary member of the Illinois State Dental Society.

The motion prevailed, and Dr. Keely was declared to be unanimously elected.

The discussion of the subject of mechanical dentistry was then resumed.

Dr. Keely considered that all Dentists were deeply interested in this question of artificial or mechanical work. He believed that Allen's continuous gum work was the best possible substitute they could employ. It possesses beauty, strength, durability and every virtue. The speaker believed that the whole country was under obligations to its inventor, and was proud to acknowledge that gentleman as his preceptor. In regard to the construction of partial sets of teeth, Dr. Keely believed that gold was decidedly the best base, though in many cases strength and a better fit can be obtained with rubber than any other substance. In the matter of full sets, too, many Dentists are too careless in obtaining the natural articulation; that is, in getting the teeth to lock in as they ought to. He considered that the Dentist who was not careful in this particular failed to perform his duty.

### RUBBER NOT SUCCESSFUL.

Dr. Kilbourne, of Aurora, said that his experience in the use of rubber in partial sets had not been flattering in any instance. In one, two or three years, a crack occurred near the first molar, and from that time the plate might be considered to be destroyed. He had had much trouble with these partial under-plates. At present, he takes all his impressions with Plaster of Paris, and gets up the dies in the same manner that he would for metal plates. He then fits a gold plate to the inside of the under front teeth, and attaches the rubber to that as a base for three or four more artificial teeth.

The speaker stated that he makes but very few rubber partial under-plates any way, and as for partial upper-plates he rarely, if ever, makes them on rubber. An exception to this rule is only made when there are four remaining molars left, two of each teeth on each side. In such instances, he uses rubber with the collar of gold plate round the first molar. The Doctor, in conclusion, expressed his belief that in general, gold is the best base for artificial dentures.

### PRACTICAL REMARKS.

Dr. Allport, for the past ten years, had done little in the mechanical part of Dentistry. Of course, like every other practitioner who had the responsibility of an office, it had fallen to his lot to advise and direct in the premises, but he had of late done but little of the actual mechanical work. In relation to partial lower cases, he thought that all that was necessary to get a good impression of the mouth is to use plaster. It does not give any more trouble, if as much, with the lower partial cases, than with any others, if only done accurately. The difficulty is, that subsequently the gum may settle, or the teeth become too short, by the absorption of the *alveolus*. In such instances, all that can be done is to make a new case, for when the gum is absorbed the teeth are not useful, by reason of their inability to touch antagonizing teeth. In full sets, no work can compete with Allen's Continuous Gum. The only objections made to such sets is their weight, but this will speedily be unnoticed; and secondly, to break if allowed to fall. This difficulty will be obviated by the exercise of care in washing them. Out of every ten cases broken, nine are brcken by being thrown down or roughly handled while being washed. Some cases, manufactured by the speaker of this material ten or twelve years ago, have been worn ever since. He believes them to be less troublesome than those of rubber or gold. The teeth rarely break in biting.

In conclusion, Dr. Allport dwelt upon the necessity by the operator in this work of the exercise of taste in color and arrangement, otherwise the rubber or gold base will prove to be as advantageous.

A great consideration in the selection and arrangement of artificial teeth is to make them look natural. Artificial teeth should not be handsome or peculiarly homely, but should be made to harmonize with the entire face, in width, color, size, position, etc. They should be selected with care, and arranged harmoniously, so as not to call the attention of a spectator to their existence upon the first glance at the face of the wearer.

There being no further discussion of this subject, Dr. J. Ward Ellis moved that it be declared passed.

The motion prevailed.

### SUSPENSION OF THE RULES.

Dr. Kennicott moved a suspension of the rules in order to appoint a committee to prepare a code of ethics.

The motion prevailed.

### DENTAL ETHICS.

Under the suspension of the rules, Dr. Kennicott moved the appointment, by the chair, of a committee of three, to prepare a code of ethics for the Illinois State Dental Society, and report as soon as practicable.

The motion prevailed, and the chair appointed as such committee, Dr. Kennicott, of Chicago; Dr. Allport, of Chicago, and Dr. Kilbourne, of Aurora.

### ADJOURNMENT.

Dr. Cushing moved that when the Society adjourns, it adjourn to meet on the second Tuesday of May next.

The motion prevailed.

The Society then proceeded to the consideration of the next subject in order, to-wit:

### THE IRREGULARITIES OF TEETH.

Dr. Keely, of Ohio, considered that the subject of irregularities of teeth was one which was too much neglected by the profession. Nineteen out of every twenty practitioners, when they saw an irregular and distorted tooth, did not attempt to place it in a right position, and considered that such an attempt would be impracticable. The speaker knew that such an opinion was fallacious, and that the operation could be successfully performed. Of course, the younger the patient was, the easier it would be to operate successfully; yet the speaker would not hesitate to move a tooth for a person thirty or forty years of age. The great virtue needed to obtain success was perseverance. If the operator would only exercise this virtue, the results would be most satisfactory. Dr. Keely, in conclusion, mentioned several difficult cases of this nature which had occurred in his own practice, in which, by the use of the wedge and inclined plane, he had gradually succeeded in bringing each refractory tooth to a proper position.

The Committee on Dental Ethics reported a Code, which was unanimously adopted.

Dr. Allport moved the appointment by the Chair of a committee of three, to confer with a similar committee of the Chicago Dental Society, with a view to merging the two into one, to be called the Illinois State Dental Society. The motion prevailed, and the committee consists of Drs. Allport, Noble and M. S. Dean.

On motion of Dr. Cushing, a committee of three was appointed to arrange subjects for discussion at the next meeting; and to adopt such means as are advisable to secure a full attendance at the next meeting. The committee consists of Drs. Kennicott and Noble, of Chicago, and Kilbourne, of Aurora.

On motion, it was voted to hold the next meeting at Chicago.

Adjourned *sine die.*

